# Comparing Virtual and In-Person Implementation of a School-Based Sexual Health Promotion Program in High Schools with Large Latino Populations

**DOI:** 10.1007/s11121-023-01526-0

**Published:** 2023-06-23

**Authors:** Bianca Faccio, Alison McClay, Krystle McConnell, Christopher Gates, Jane Finocharo, Julia Tallant, Valerie Martinez, Jennifer Manlove

**Affiliations:** 1 Child Trends, 7315 Wisconsin Ave, Ste 1200W, Bethesda, MD 20814 USA; 2 The University of Maryland, College Park, MD USA; 3 Identity, Inc, Gaithersburg, MD USA

**Keywords:** Program implementation, Teen pregnancy prevention, Program evaluation, COVID-19, Sexual health promotion, Latino youth, Positive youth development program

## Abstract

Many sexual health programs transitioned to virtual implementation during the COVID-19 pandemic. Despite its devastation, the pandemic provided an opportunity to learn about virtual compared to in-person implementation of a sexual health promotion program—El Camino. This study assessed differences in program attendance, engagement, quality, and student ratings for virtual versus in-person implementation of El Camino as part of a rigorous evaluation in high schools with high Latino populations in Maryland. Drawing on positive youth development practices, El Camino helps participants identify personal goals and learn about sexual reproductive health and healthy relationships. This mixed-methods study incorporates data from performance measures, baseline and post-intervention participant surveys, observations, monthly implementation reports, and debriefs with facilitators to describe and compare virtual and in-person program implementation. At baseline, participants were an average of 16.2 years old; between 8 and 12^th^ grade; 61% female; 79% Hispanic, Latino, or of Spanish origin; and 54% spoke mostly Spanish at home. Recruitment and retention of students outside of school classes were challenging for both forms of implementation. However, attendance was higher during in-person implementation and in schools where the organization implementing El Camino had a strong presence before the pandemic. Findings indicate high fidelity, excellent quality ratings, and positive student perceptions of the program and facilitators in both the virtual and in-person cohorts, which suggest that both forms of implementation were comparable and furthermore highlight the strength of the virtual adaptation of the El Camino program.

The COVID-19 pandemic was linked to declines in school attendance, increases in mental health issues among adolescents, and spikes in school failure, particularly among those most vulnerable (Cockerham et al., 2021; Centers for Disease Control and Prevention [CDC], 2022; Office for Civil Rights, 2021). During the pandemic, Latino youth and English language learners were especially likely to live in families that experienced economic, physical, and mental health hardships that could affect school engagement (Padilla & Thomson, 2021). Although maintaining or increasing knowledge gains when transitioning from in-person to virtual implementation was possible, organizations experienced several disadvantages during virtual implementation, including efforts to adapt curricula for virtual implementation, time constraints, difficulties building relationships in a virtual environment, and maintaining student engagement in programming outside of regular school classes or activities (Fernandez et al., 2021; Ogletree & Bey, 2021). Existing research specifically examining the adaptation of sexual health curricula to virtual implementation primarily focuses on its reception among youth participants, finding it to be generally positive (Patel et al., 2022). The current study expands previous research by examining facilitator experiences delivering a sexual health curriculum among participants largely comprised of Latino youth and English language learners in addition to participants’ ratings of the program and its mode of implementation.

El Camino is an 11-lesson research-based and positive youth development–informed sexual health promotion curriculum designed for adolescents, particularly Latino youth (Child Trends, 2021a). Originally developed for in-person implementation, the program was adapted for virtual implementation in Fall 2020 in response to the pandemic. As part of a randomized controlled trial, Child Trends (program developer) partnered with Identity, Inc. (Identity; implementation partner) and the University of Maryland (UMD; independent evaluator) to implement El Camino in high schools with large Latino populations in Montgomery County, MD, beginning in February 2021 until early 2023. The pandemic worsened educational disparities for Black, Latino, and low-income students in Montgomery County Public Schools [MCPS] (MCPS, n.d.; Lewin & Roy, 2020), and there was a marked increase in the proportion of MCPS students with limited English proficiency who received failing grades during remote learning (St. George, 2020). Furthermore, MCPS students wanted and needed regular interaction with adults who could provide support as well as frequent and more effective communication (Lewin & Roy, 2020).

This study describes and compares virtual and in-person implementation of El Camino and explores how program delivery during the pandemic impacted both forms of implementation by providing information about participant and facilitator experiences. This study uses data from the first three cohorts: Spring 2021 (virtual, February–May 2021), Summer 2021 (in-person, July 2021), and Fall 2021 (in-person, October 2021–January 2022). We used quantitative data from participants’ baseline and post-intervention surveys, observer reporting, student attendance; and qualitative data from facilitators’ fidelity logs, monthly reports from Identity’s program manager, and notes from discussions with facilitators.^1^

## Methods

### Intervention

The El Camino evaluation study was reviewed and approved by the Child Trends Institutional Review Board (FWA00005835) and started in February 2021 and is ongoing through 2023. El Camino can be implemented in both English and Spanish and consists of eleven 45-min lessons divided into three sections (or arcs) that focus on goal setting, sexual and reproductive health, and healthy relationships (see Table 1). Throughout the curriculum, youth are encouraged to identify and set goals, make informed reproductive health choices, and have healthy relationships (Child Trends, 2022). Child Trends collaborated with curriculum writers, Identity, and UMD to adapt, pilot, and revise the curriculum for virtual implementation while preserving the core components^2^ of the in-person curriculum (Parekh et al., 2021) (see Table 2).

**Table 1:** A Summary of El Camino curriculum lessons

**Arc 1: Goal Setting**	
**Lesson 1: State Your Goal: Intro to El Camino.** Students learn about setting and achieving their goals via the **STAR** steps and identify a goal for themselves at age 25	**Lesson 2: Think About the Steps: My Life at 25.** Students identify steps they will need to take to achieve the goal they identified in Lesson 1 as well as learn of tools and resources to help them achieve their goal
**Lesson 3: Assert Your El Camino: Dating & Decisions about Sex.** Students discuss positive and negative road trips that can affect their *camino*	**Lesson 4: Reach Your Goal: Setting Limits to Stay on Track.** Students discuss warning signs and how road trips can affect their *camino*
**Arc 2: Sexual and Reproductive Health**	
**Lesson 5: Teen Pregnancy and Understanding How a Pregnancy Occurs.** Students describe basic reproductive anatomy and learn important facts about pregnancy	**Lesson 6: Promoting Sexual Health: Contraception.** Students learn about safe and effective contraception, discuss how using contraception can help protect their *camino*, and describe how a person can support their partners in using hormonal contraception
**Lesson 7: Promoting Sexual Health and Preventing STIs: Condoms.** Students describe the benefits of correctly and consistently using condoms, learn how to use an external condom and about STIs, and describe the roles both partners can play in using condoms	**Lesson 8: Promoting Sexual Health and Staying on Your Camino.** Students build on the knowledge and skills gained and discuss ways to feel more comfortable talking about contraception with a health care provider and discuss how using condoms and contraception can help them protect their *camino*
**Arc 3: Healthy Relationships**	
**Lesson 9: Assertive Communication: Setting and Protecting Our Personal Limits – Part 1.** Students learn how to set, communicate, and protect limits regarding sex with partners; define passive and assertive communication; and describe how limit setting is part of a healthy relationship	**Lesson 10: Assertive Communication: Setting and Protecting Our Personal Limits – Part 2.** Students explain the characteristics of consensual sex and use assertive communication to set and maintain limits regarding sex and protected sex in skits
**Lesson 11: El Camino and Your Future.** Students apply all they have learned and revisit their El Camino Goal Map and practice setting, communicating, and maintaining limits regarding sex and protected sex with partners

**Table 2:** Activity and implementation differences for the El Camino curriculum

	In-person	Virtual	Preserving core components
Activity			
General implementation	No Power Points	Power Points	Curriculum continued to be youth focused
Vote with your feet	Physically moving from one end of a classroom to another end of the classroom	Using chat box, reactions, or voice to vote	Facilitators continue to not tell the youth what to think, believe or do
Pass the ball	Throw a ball	Call out another student by name	Youth continue to identify positive goals for their future and ways to achieve them and continue to utilize the STAR goal-setting model
Pedro’s party	Clap, snap, or raise your hand when there is a warning sign	Use reactions when there is a warning sign	Youth continue to learn about healthy relationships, consent, and develop assertive communications skills
Question box	Use post-its or index cards to write questions anonymously	Virtual box via Google Forms	Youth continue to have the right to accurate information about reproductive biology and effective methods of contraception to help them to avoid unintended pregnancy and STDs
How a pregnancy occurs	Lecture, handout, questions	Shortened lecture content and videos (first show photo, then show videos, then short lecture, then complete handout, and finish with questions)	Youth continue to learn about reproductive health, healthy relationships, and resources (people, institutional, economic) that could assist youth as they work toward their goals and learn about
Condom demonstration	Use penis models	Use videos and substitute condoms for a sock	Youth continue to have the right to accurate information about effective methods of contraception to help them to avoid unintended pregnancy and STIs
Skits	Divide students into small groups of 3–4 students each. All students practice skill(s) multiple times	Divide students into two breakout groups. All students get to practice skill(s) at least once	Facilitators continue to not tell the youth what to think, believe, or do
Homework	Students are asked to have a conversation with a parent, guardian, or trusted adult about a topic relevant to the curriculum	No change	Parents, guardians, and trusted adults continue to be engaged to support youth to reach their education and family formation goals
Implementation			
Timing	Lunchtime	Study period	
Setting	Classroom	Zoom meeting	

The current study took place in nine^3^ high schools with large Latino, including Spanish-speaking, populations in Montgomery County, MD. Three of the implementation sites took place at MCPS high schools with School Based Health and Wellness Centers (Wellness Center schools), which Identity operates and has an established relationship with the school and student community. The remaining five schools were the result of new partnerships Identity established for the study (non-Wellness Center schools). Each school recruited students to participate in one of two groups (5–18 students per group) that was to be randomly assigned. Parental consent and student assent were obtained prior to student enrollment into the study. Once enrolled students completed the baseline survey, groups were randomly assigned to receive either the intervention curriculum (El Camino) or a different, unrelated leadership development program of the same length (11 lessons) called PODER. Randomization of the condition was altered by semester. Each curriculum was delivered by two trained facilitators, and implementation was conducted in English and Spanish (as appropriate) as well as virtually and in-person. For the Spring 2021 and Fall 2021 cohorts, most schools implemented El Camino once a week, although there were some exceptions when implementation occurred twice a week. The Summer 2021 cohort was implemented during a 2-week period and was notably smaller (*n* = 19).^4^

### Data Collection and Analysis

#### Quantitative Data

Student survey data were collected at baseline prior to randomization and post-intervention immediately following program implementation. This paper focuses on the experiences of 185 students (17 classes) who received El Camino (76 students in 8 classes during the Spring 2021 virtual cohort; 109 students in 9 classes during the Summer 2021 and Fall 2021 in-person cohorts). Surveys were self-administered and included questions on student demographics, psychological distress, and student ratings of the program and facilitators. Psychological distress was assessed using the six-item Kessler Screening Scale for Psychological Distress (K6; Kessler et al., 2002), which has been validated in youth samples and shown to have excellent internal consistency reliability (Cronbach’s *α* = 0.89). The K6 is scored using a 5-level response scale, ranging from 0 (none of the time) to 4 (all of the time), which generates a scoring scale with a range of 0 to 24. Study data were collected and managed using REDCap electronic data capture tools hosted at Child Trends (Harris et al., 2009, 2019). Participants received a $10 gift card for completing each survey and for each session they attended. Survey procedures were the same for virtual and in-person implementation.

Facilitators monitored attendance and tracked the percentage of students who attended at least 75% of lessons (9 out of 11 sessions) as a performance measure. Trained observers from Child Trends and UMD (*n* = 15) observed 29 sessions across the three cohorts. Prior to conducting an observation, all observers completed an annual training on conducting culturally responsive observations provided by UMD. Each implementation site was observed at least once, and observations were spread throughout the implementation period to ensure that a variety of lessons were observed. Observers completed an observation form via REDCap, which included questions with corresponding Likert scale responses to assess student engagement, facilitator qualities, and overall quality of the session, which were reported as performance measures. Observers noted different actions indicating student engagement between virtual and in-person implementation. For virtual implementation, engagement included instances of a student turning on their camera, verbally sharing, using the chat, or using the reaction feature, whereas for in-person implementation, engagement included students verbally sharing, following the lesson in their workbook, participating in activities, and completing worksheets.

#### Qualitative Data

Facilitators completed fidelity forms (*n* = 182) via REDCap after every lesson to monitor how closely their program delivery adhered to the written program. Facilitators were trained on the importance of fidelity and how to complete the fidelity logs prior to implementation (Child Trends, 2021b). Facilitators provided open-ended feedback whenever they indicated activity adaptations or noncompletion and gave additional feedback as relevant. Child Trends conducted debriefs (*n* = 6) with facilitators after lessons 4, 8, and 11 during the Spring and Fall 2021 semesters. During these debriefs, facilitators reflected on the successes, challenges, and overall experiences implementing each curriculum arc, and exchanged strategies related to engagement and retention. Monthly reports (*n* = 13) were written by Identity’s program manager with input from facilitators and included information about activities completed and successes and challenges encountered. The reports were informed by conversations and email updates from facilitators about implementation in their respective schools.

#### Quantitative Analysis

We assessed statistically significant differences between virtual and in-person participant baseline characteristics, attendance, and post-test program ratings using two sample *t*-tests for continuous measures and chi-squared tests for categorical measures. We conducted significance tests of student program ratings by implementation type (virtual vs. in-person), controlling for grade level, length of time in the USA, and whether they ever had sex. Control variables were selected due to observed differences in baseline responses between implementation type. We assessed statistically significant differences in student attendance of 75% or more lessons by implementation setting and school type (Wellness Center vs. non-Wellness Center) using the chi-squared test of independent proportions. Data from observation forms were used to calculate the mean observed quality ratings by implementation type. Statistical significance was not tested due to small sample sizes. All analyses were completed using SAS Studio [v5.4, 2019].

#### Qualitative Analysis

Monthly reports, facilitator debrief notes, and responses to open-ended questions from the facilitator logs were transferred into Excel for thematic analysis (Maguire & Delahunt, 2017). Two trained researchers coded the data and identified commonalities across the three data sources, which included attendance, incentives, student engagement, staffing/capacity, uncertainty amid the pandemic, working with school partners, changes in modality, and fidelity. Through extensive discussion, the coders agreed upon the larger themes presented in this paper.

## Results

### Student Characteristics

A self-administered baseline survey showed that over half the participants in virtual and in-person implementation (61.4%) were female and on average 16.2 years old (see Table 3). Most (79.0%) participants were of Hispanic origin and either spoke mostly Spanish at home (54.4%) or both Spanish and English at home (21.7%). Almost one quarter (22.9%) of the sample had ever had sex, and 9.7% had sex in the last 3 months. On average, a marginally lower percentage of in-person participants ever had sex than virtual participants. There was no difference in psychological distress reported between in-person compared to virtual participants, with a mean K6 score of 13.4 in both groups. The overall mean K6 score of 13.4 is higher than what has been reported in general adolescent samples in the USA (Mewton et al., 2016; Peiper et al., 2015). Compared with virtual participants, in-person participants were younger. Additionally, a higher percentage of virtual students reported having recently arrived to the USA (< 3 years). Finally, virtual participants (94.7%) were more likely to be in 9^th^ to 11^th^ grade than in-person participants (82.6%).

**Table 3:** Student baseline characteristics, by implementation setting

Student characteristics	Virtual	In-person	Total	*p*-value
*N* (at baseline)	76	109	185	
Age (average)	16.5	16.0	16.2	0.048*
Grade				0.014*
9^th^–11^th^	94.7%	82.6%	89.1%	
Gender				0.310
Female	55.3%	65.1%	61.4%	
Race/Ethnicity				0.994
Hispanic, Latino, or Spanish Origin	79.0%	78.9%	79.0%	
Language spoken at home				0.681
Mostly Spanish	56.0%	53.2%	54.4%	
Mostly English	17.3%	21.1%	19.6%	
Both Spanish and English	24.0%	20.2%	21.7%	
Other	2.7%	5.5%	4.3%	
Time in the USA				0.017*
Born in the USA	29.6%	35.7%	33.1%	
< 3 years	49.3%	28.6%	37.3%	
> 3 years	21.1%	35.7%	29.6%	
Ever had sex	30.1%	17.9%	22.9%	0.056
Had sex in the last 3 months	12.7%	7.6%	9.7%	0.265
Psychological distress in the last 30 days (average)	13.40	13.40	13.40	0.999
Nervous	47.4%	55.0%	51.9%	0.304
Hopeless	36.8%	30.3%	33.0%	0.350
Restless or fidgety	32.9%	34.9%	34.0%	0.781
Depressed	33.0%	24.7%	28.1%	0.227
Everything an effort	43.4%	47.7%	46.0%	0.565
Worthless	25.0%	24.0%	24.3%	0.858

^*^p < .05

### Program Implementation Findings: Comparing Virtual and In-Person Implementation

Our analysis identified three main themes: recruitment, attendance, and student engagement. Notably, all aspects of program implementation were challenging during the pandemic for both virtual and in-person cohorts.

#### The Pandemic Presented Challenges to Recruitment for both the Virtual and In-Person Cohorts

Staff at Identity described challenges in identifying and reaching potential participants in a virtual setting. Recruitment was conducted via Zoom or email, and school partners reported overall low engagement and attendance of students at prospective information sessions. Identity staff shared, “COVID-19 devastated our client community as [a] disproportionate number of partners and caregivers lost jobs and became ill or both. Students who were already challenged are struggling to succeed with remote schooling.” As a result, Identity expanded recruitment beyond the initially targeted classrooms to reach an ideal enrollment of at least 10 students per classroom and extended the amount of time scheduled for recruitment to at least three weeks.

After returning to in-person implementation for the Summer and Fall 2021 cohorts, schools limited in-person time for students, parents, and outside program providers to reduce the risk of COVID-19 exposure. Identity had limited access to activities that would normally aid recruitment, such as new student orientation, back to school nights, and regular school class visits. The program manager reported, “Due to the restrictions that have resulted from the pandemic, staff has limited ability to devote time to engaging students, building relationships, and checking in with them face-to-face.” They also shared that recruitment, including time needed to consent participants, required more time and staff than originally allocated.

#### Attendance Was Higher in Person and Among Wellness Center schools, Which Had Existing Relationships with the Implementation Partner

While facilitators made substantial efforts to increase and maintain attendance, student attendance was significantly higher for in-person (45.8%) than virtual implementation (29.0% attended 75% or more lessons; see Table 4). This difference was driven by higher in-person (77.6%) than virtual attendance (33.3%) in Wellness Center schools.^5^ In contrast, attendance among students in non-Wellness Center schools was lower than 30% for both virtual (25.6%) and in-person (19.0%) implementation. Across cohorts, facilitators expressed that attendance was higher when students in classes knew each other and were friends, which was more challenging in virtual implementation.

**Table 4:** Program attendance, by implementation setting and school type

School type	Total		Virtual		In-person		*p*-value
	Total # participants	Attended 75% or more lessons	Total # participants	Attended 75% or more lessons	Total # participants	Attended 75% or more lessons	
Wellness Center schools (*n* = 3)	82	59.8% ^a^	33	33.3%	49	77.6% ^a^	< .001**
Non-Wellness Center schools (*n* = 5)^b^	101	21.8%	43	25.6%	58^c^	19.0%	0.426
Total (*n* = 8)	183	38.8%	76	29.0%	107	45.8%	0.021*

We conducted significance tests of student attendance of 75% or more lessons by implementation setting (virtual vs. in-person) using chi-squared tests of independent proportions

^a^Attendance in Wellness Center schools is significantly different from attendance in non-Wellness Center schools (*p* < .001)

^b^One of the non-Wellness Center schools that participated in the Spring 2021 cohort was replaced with a different school in Fall 2021. Thus, a total of nine schools (including 6 non-Wellness Center schools) were engaged throughout the study period with a maximum of eight schools (and 5 non-Wellness Center schools) represented in each cohort

^c^Attendance data from the Fall 2021 cohort is missing for two non-Wellness Center school participants; thus, the total sample for attendance is two less than the full sample (*n* = 185)

^*^ *p* < .05. ** *p* < .001

In general, student attendance was significantly higher in Wellness Center schools than other schools (59.8% vs. 21.8% attended at least nine of 11 sessions). The program manager attributed the higher attendance at Wellness Center schools to existing relationships with school partners and students: “Many of the students recruited had a personal relationship with Identity staff or were part of a class where the teacher worked closely with Identity staff and were intimately familiar with programs available.”

During virtual implementation, most El Camino lessons were held on weekdays when students were not in school classes. Lower attendance during virtual implementation was due in part to other student responsibilities, such as needing to provide childcare, work, or illness. Program staff offered flexible options, such as make-up sessions for students who could not attend scheduled sessions. However, Zoom fatigue (Peper et al., 2021) challenged program planning, delivery, and implementation. The program manager shared:

Since Identity operates as voluntary, extracurricular programming, much of what Identity does has become an additional effort and demand, in some cases. Pre-pandemic, teachers, school staff and students alike showed more enthusiasm for after school extracurricular activities. During these times, … they are ready to check out.

During in-person implementation, most El Camino sessions were held during lunch in both types of schools, which also caused challenges. Students often spent their lunch periods meeting with teachers or guidance counselors, making up assignments, taking tests, or utilizing their free time. Facilitators also noted that lunchtime implementation in non-Wellness Center schools was challenging due to student unfamiliarity.

To maintain regular student attendance during virtual and in-person implementation, facilitators sent reminders to participants via text or called the morning of each session. These reminders were particularly important when the school district began allowing students to opt-in to in-person instruction during the Spring 2021 cohort, and to ensure facilitators provided an appropriate amount of food for participants during in-person implementation.

#### Student Engagement Looked Different Across Implementation Modalities

Facilitators shared that engaging youth was crucial for both attendance and participation. They also noted key differences in student engagement based on whether implementation occurred virtually or in-person. For both implementation modes, facilitators used many different methods (as described above) to stay connected with participants between lessons and throughout implementation. Facilitators stated that the participants who attended lessons became more engaged as time progressed if they had friends in the group or during more interactive lesson activities, such as skits and role playing. One facilitator noted that some participants were “apprehensive about discussing or opening up but as [the] group went on and they felt comfortable, they started to open up.” Another facilitator shared, “we are seeing the students participate more and more as the lessons got more intense. They use their voice more, participate well with one another. [I’m] excited to see how the next weeks will go as the lessons become more interactive.”

Student engagement looked different virtually versus in-person as did the measurement criteria for student engagement during virtual implementation, accounting for differences in lesson delivery. During virtual implementation, facilitators shared that building rapport as well as engaging students required more time, patience, effort, and creativity than past experiences with in-person implementation (Parekh et al., 2021). In fidelity logs, facilitators reported difficulty engaging students in discussion in virtual sessions, particularly in the beginning of the semester. One facilitator reported there was “low student participation” in Lesson 1, and another facilitator said “participation is still a little slow and minimal” for Lesson 2. Facilitators noted that students used Zoom’s chat and reaction features more than their camera or microphone. Facilitators actively thought of ways to better engage students in the first few lessons, including encouraging (but not requiring) cameras to be on, asking students to elaborate on their feedback in the chat, or reacting to other students’ responses to create more space for participation. Facilitators noted that student engagement in virtual sessions improved as the program went on, perhaps due to increased comfort with other students in their class or the program itself. For example, one facilitator wrote in their fidelity log for Lesson 7: “The kids were very engaged in this lesson. It was challenging to have this important conversation over Zoom because the kids are around their family sometimes but given the challenges people participated well.”

When implementation returned to in person, facilitators reported that students seemed more engaged but noted that this could be due to ease in assessing engagement when everyone is in the same room. Facilitators shared that in-person participants were quicker to respond during group discussions, appeared interested, asked questions, and seemed excited to be back in-person. The program manager reported, “Program facilitators noted a stark improvement from virtual delivery to this in-person group in participation… Besides facilitators observing increased participation, there was a noticeable increase in use of the question drop-box use from participants.” While lunchtime implementation had low attendance at times, students who attended were highly engaged. The program manager shared, “By all accounts from facilitators, engagement and participation by students is high. Participants attending the groups are interested, interactive and responsive to activities and questions during session.”

Despite differences in how students engaged with the curriculum, observation ratings of overall student engagement were similar across cohorts. On a scale of 1 (less than 25% of participants) to 5 (75–100% of participants), most students actively participated in discussions and activities (4.0 for virtual vs. 4.6 for in-person) and appeared interested (4.0 for virtual vs. 4.6 for in-person). Observers also reported that facilitators had similar above average to near excellent levels of enthusiasm, rapport, and communication with participants, regardless of implementation setting (see Table 5). Additionally, while student participation was challenging in virtual settings, students in both groups rated the program and facilitators highly and gave El Camino high-quality ratings at post-test. The majority of virtual (88.9%, see Table 6) and in-person (92.1%) students rated the program as excellent or very good. More than nine in 10 students liked the facilitators (96.6% virtual and 96.0% in-person). Virtual (68.8%) and in-person (80.0%) students also reported that they learned a lot. In-person participants (89.3%) reported that discussions helped them learn more often than virtual participants (77.8%); however, this difference was not statistically significant difference at the 0.05 level and was thus only marginally higher (*p* = 0.065). While student engagement was challenging for both virtual and in-person implementation, experience and quality were comparable across both modalities.

**Table 5:** Mean observed quality ratings, by implementation setting

	Virtual	In-person
***N***	**11**	**18**
Facilitator qualities		
Teacher’s explanations of activities	4.6	4.8
Keeping track of time	3.6	4.3
Presentation being rushed or hurried	4.2	4.3
Student engagement		
Appear to understand the material	4.7	4.6
Participate in discussions and activities	4.0	4.6
Interested in lessons and activities	4.0	4.6
Implementer rating based on the following: knowledge, enthusiasm, poise and confidence, rapport and communication, effectively addressing questions	4.4	4.6
Overall quality	4.2	4.4

We did not test significance due to small sample size

**Table 6:** Student post-test ratings of the program and facilitators, by implementation type

	Virtual	In-person	*p*-value
***N***	64	76	
Overall rating of the program: excellent or very good	88.9%	92.1%	0.517
Liked facilitators	96.6%	96.0%	0.853
Learned a lot	68.8%	80.0%	0.128
Discussions helped them learn	77.8%	89.3%	0.065

## Discussion

This paper expands previous research by comparing successes and challenges for in-person and virtual implementation of the El Camino sexual health promotion program. Using data from student surveys, facilitator and observer logs, and facilitator and program reports, we found higher attendance during in-person vs. virtual programming, but similarly high observer quality and student ratings of the program facilitators in both implementation modes. We also identified challenges with recruitment and engagement in both implementation settings.

Maintaining high recruitment and attendance can be a challenge with voluntary student programming outside of regular classes (Afterschool Alliance, 2009). Overall, we found lower attendance in virtual than in-person classes, which has been found in other studies (Goldstein et al., 2020; Meeter et al., 2020; Weijers et al., 2022). However, the implementation team noted unique challenges with recruitment and attendance during the pandemic for both virtual and in-person implementation. The high levels of student mental health issues in our sample echoed national reports during the pandemic (CDC, 2022) and may have reduced student engagement in programming. For in-person programming, program staff had limited access to the schools for recruitment, and many schools limited after-school activities. For virtual programming, students also faced Zoom fatigue (Peper et al., 2021) after participating in a full day of virtual school.

Previous research has highlighted relationship-building between facilitators and students as key to encourage student recruitment, attendance, and engagement (Luo et al., 2017). The dramatic differences in recruitment and attendance for schools in which the facilitators already had a presence (Wellness Center schools) were striking. They had especially high attendance relative to other schools during in-person implementation. This finding supports the benefit of having a physical presence in the school, outside of the specific after-school program.

While facilitators described difficulty engaging students virtually in early lessons, high student and observer ratings of the program and facilitators suggest that a well-developed adaptation of a sexual health curriculum can be feasible and high quality.^6^ Recent research has described best practices for transitioning to virtual implementation, such as using interactive activities; using a platform that allows participants to virtually raise their hands; having two facilitators with one monitoring platform logistics and the other focusing on program content and engagement; having live sessions to encourage interaction between students and facilitators; and having a variety of ways for students to engage in virtual programming (such as using the chat or reactions) to allow for more voices in group-based discussions (Caprara & Caprara, 2022; Domina et al., 2021; Ogletree & Bey, 2021; Parekh et al., 2021; Sweetman, 2020). Beyond the pandemic, virtual programming can reach students in states that do not have such programs or students who are unable to attend programs locally. Some of these alternatives may also benefit in-person programming, especially for students who are introverted or are uncomfortable speaking in class (Callahan, 2021; Tuovinen et al., 2020; Yu, 2021).

High observer ratings of student engagement in virtual programming may reflect differences in how observers assessed engagement across the two types of implementation modes. Facilitators and observers noted that most students kept their cameras off during virtual implementation—as students may share space with other family members—but could reduce the ability to assess student-facilitator and student-peer engagement, which is key in in-person implementation (Parekh et al., 2021). Program staff specifically reported higher enthusiasm and greater engagement of students and facilitators in in-person programming, which aligns with other research citing lower engagement in virtual programming (Domina et al., 2021)**.**

## Limitations

There were several limitations to this research due in part to the changes in school policies in response to the pandemic. During the Spring 2021 cohort, students were given the option to return to in-person learning midway through implementation (MCPS, n.d.), so many students returned to school in-person, which affected program attendance. For the Fall 2021 cohort, a surge of COVID-19 cases in December 2021 forced all extracurricular activities online, including El Camino, which resulted in attendance being lower than expected for the last 2–3 lessons. The team also had difficulty gathering student input via focus groups to learn more about their experiences participating in this program during the pandemic, so we only have student input from post-test surveys. Because we only analyzed participants that received the El Camino curriculum during the pandemic, our sample size is relatively small, which limits our ability to detect differences. Finally, we did not incorporate a comparison of implementation modality into the evaluation study design and instead conducted ad hoc analyses of data collected during the pandemic. However, these limitations were offset by rich information available to compare virtual and in-person implementation.

## Conclusion

Conducting an evaluation during the COVID-19 pandemic allowed us to compare virtual and in-person implementation. While attendance was lower in virtual than in-person implementation, we found high fidelity and positive observer and student ratings of the program and facilitators in both implementation modes, highlighting the strength of the in-person and virtual implementation. In future research, we plan to compare the impacts of virtual with in-person implementation of El Camino. Additional evaluations should assess the efficacy of virtual sexual health program implementation post-pandemic, which could ultimately expand the types of programming available to youth living in low-resource areas.

## Footnotes

1 Facilitators are the people who implement the El Camino program. Facilitators either work for or are contracted by Identity, Inc., our implementation partner.

2 Core components of the program are summarized in Child Trends. (2021a, 2021b). *El Camino: A goal-setting sexual health promotion curriculum. Key Background Information for Implementing El Camino*. https://www.childtrends.org/wp-content/uploads/2021/04/ElCaminoFrontMatterAppendices_ChildTrends_June2021.pdf.

3 Eight schools (3 Wellness Center, 5 non-Wellness Center) participated in Spring 2021; 1 school (Wellness Center) participated in Summer 2021; and 8 schools (3 Wellness Center, 5 non-Wellness Center) participated in Fall 2021. One of the non-Wellness Center schools that participated in the Spring 2021 cohort was replaced with a different school in Fall 2021. Thus, a total of nine schools were engaged throughout the study period with a maximum of eight schools represented in each cohort.

4 Given the potential of the shorter implementation period of the Summer 2021 cohort to impact curriculum experience and quality, sensitivity analysis excluding the Summer 2021 cohort was conducted. Findings were remarkably consistent.

5 Sensitivity analyses found that there were no significant demographic or sexual behavior differences between virtual and in-person attendees among Wellness Center schools.

6 Based on our lessons learned, we have published strategies to help facilitators engage students virtually. This resource can be found via Child Trends. (2021b). *El Camino: A goal-setting sexual health promotion curriculum—Key background information for implementing El Camino.* https://www.childtrends.org/wp-content/uploads/2021/04/ElCaminoFrontMatterAppendices_ChildTrends_June2021.pdf
